# General practitioners and palliative care practices: a better knowledge of specific services is still needed

**DOI:** 10.1186/s12913-024-11266-8

**Published:** 2024-07-23

**Authors:** Daouda Niaré, Guillaume Robert, Auriane Rocquevieille, Loïc De Geyer, Maguy Frin, Sophie Pennec, Thomas Hanslik, Thierry Blanchon, Louise Rossignol, Vincent Morel

**Affiliations:** 1 Sorbonne Université, INSERM, Institut Pierre Louis d’Épidémiologie et de Santé Publique, IPLESP, F-75012 Paris, France; 2 Centre Hospitalier Universitaire (CHU) de Rennes, Service de soins palliatifs, 35033 Rennes Cedex, France; 3 Université Rennes 1, Rennes, France; 4 Département de Médecine Générale, Université Paris Cité, F-75018 Paris, France; 5 Institut National d’études Démographiques (INED), F-93320 Aubervilliers, France; 6 School of Demography, Australian National University, Canberra, Australia; 7 UFR des sciences de la santé Simone-Veil, Université de Versailles Saint-Quentin-en-Yvelines, F-78180 Montigny-le-Bretonneux, France; 8 Service de médecine interne, Hôpital Ambroise-Paré, Assistance publique–Hôpitaux de Paris, AP-HP, F-92100 Boulogne Billancourt, France; 9 Université de Rennes, INSERM, Centre d’investigation clinique de Rennes (CIC 1414), 35000 Rennes, France

**Keywords:** Palliative care, End of life care, Physicians, Primary health care, Knowledge, Professional practice, Home care services

## Abstract

**Background:**

France allows deep sedation for pain relief, but not for euthanasia. In anticipation of an increase in home-based palliative care, the role of general practitioners is central to the design of outpatient palliative care services. This study aimed to describe the knowledge, attitudes, and practices of general practitioners in mainland France regarding palliative and end of life care.

**Methods:**

This was a national descriptive cross-sectional study within the Sentinelles network. Self-report questionnaires were distributed to general practitioners between November 2020 and November 2021. A descriptive analysis was carried out.

**Results:**

Out of the 123 participating general practitioners, 84% had received academic training in palliative care (*n* = 104). While a significant majority (69%) expressed comfort in pain management, only a quarter (25%) declared that they were competent at indicating deep and continuous sedation for pain relief. Awareness of outpatient palliative care facilities close to their place of practice such as hospitalization at home was over 97% (*n* = 117/120). Awareness of hospital facilities, including identified palliative care beds on hospital wards and palliative care units, was lower (75% (*n* = 59/79) and 86% (*n* = 86/100), respectively).

**Conclusions:**

Our results suggest that French general practitioners are reasonably aware of palliative care resources available. However, there is room for improvement, particularly in understanding hospital-based facilities. Furthermore, a quarter of the general practitioners expressed discomfort with deep and continuous sedation for pain relief, highlighting the need for increased training in this specific aspect of palliative and end of life care.

**Supplementary Information:**

The online version contains supplementary material available at 10.1186/s12913-024-11266-8.

## Key statements

### What is already know about the topic?

- General practitioners play a central role in providing home-based palliative and end of life care.
- Despite academic training in palliative care for general practitioners, several studies have reported a lack of training, knowledge, and utilization of palliative care services among general practitioners.

### What this paper adds?

- More than half of French general practitioners have received minimal training in palliative care, and a majority lack experience in palliative care services.
- Only a quarter of French general practitioners felt competent to indicate deep and continuous sedation for pain relief.
- French general practitioners were more aware and made greater use of outpatient palliative care services than palliative hospital-based services.

### Implications for research, practice and policy

- This study highlights the need for further research into the training requirements and access of general practitioners to hospital-based palliative care services.
- Our findings highlight the need for more training for general practitioners in the use of deep and continuous sedation, particularly in view of the growing elderly population and chronic diseases that require more palliative care at home. In addition, evolving end-of-life legislation in many countries worldwide underscores the importance of such training.
- Another implication is the need for increased interaction between general practitioners and providers of palliative care services, particularly hospital-based palliative care services.
- Future policies for the implementation of palliative care services should consider the distribution of these services to achieve an equitable distribution across the country.

## Background

The aging of the population is an increasing concern in many countries. A quarter of the French population is 60 years and older, with the proportion of those aged 75 and above increasing from 4.7% in 1970 to 10% in 2019 [1]. Chronic diseases, cancers, and polymorbidities are prevalent among elderly people and often necessitate palliative care, impacting patient support and end-of-life care (https://www.who.int/news-room/fact-sheets/detail/palliative-care). These elderly people receive most of their care in the community, either at home or in residential care facilities [2]. Projections in England and Wales indicate that the number of people requiring palliative care will increase by 25% by 2040 if mortality trends of 2014 continue [3]. In this context, the relationship between GPs and specialist palliative care providers is key to responding effectively to the projected increase in palliative care need [4–6].

Globally, many people prefer to spend their final moments at home [7–9]. This preference varies from 31 to 87%, reaching 100% for cancer patients, as evidenced by systematic reviews from America, Europe, Asia, Africa, and Oceania [10–12]. According to a 2014 European study, 35% of people died at home non-suddenly in Belgium, 50% in Italy, 51% in Spain and 51% in the Netherlands, and the proportions of people who died in their desired place of death were 73%, 68%, 86% and 75%, respectively, for the same countries [13]. The congruence between preferred and actual place of death is influenced by the availability of community-based resources, including home care, facilitation of hospital discharge, and management of complex health conditions [7, 9]. However, despite this desire to die at home, specialist palliative care is not available to many dying people [10].

According to the World Health Organization (WHO), palliative care is a holistic approach that provides relief to patients suffering from life-threatening diseases and supports their families throughout the course of the illness. It prevents and relieves suffering through the early identification, correct assessment and treatment of pain and other problems, whether physical, psychosocial or spiritual. WHO emphasizes the initiation of palliative care from disease onset, extending beyond terminal stages, this means that palliative care should not be perceived solely as end-of-life care. Palliative care must provide by a multidisciplinary team include doctors, nurses, support workers, pharmacists, social workers, physiotherapists and volunteers all working together with the patient and their family (https://www.who.int/news-room/fact-sheets/detail/palliative-care).

In many countries, General practitioners (GPs) play a crucial role in facilitating home-based care, especially in palliative and end of life contexts notably by providing continuity of care by setting up an individualized care pathway from diagnosis to palliation, coordinating care across that pathway, and collaborating with other health care providers [14–19]. A Dutch study identified various barriers encountered by GPs in the context of palliative care, categorized into three levels: personal (related to knowledge, skills, and emotions), relationship (related to communication with patients, other care providers, and collaboration), and organizational (related to the organization of care) [20]. To implement early palliative care effectively, it is essential for GPs to identify patients who need palliative care [21, 22]. Nevertheless, several studies have demonstrated that GPs both lack and require training in palliative and end of life care [6, 18, 23–27]. An Italian study among 1,489 GPs reveals some uncertainty on the part of Italian GPs regarding the definition and goals of palliative care [28]. In an Australian study, a significant number of GPs (31%) reported a lack confidence in providing palliative care because of patient complexity, inadequate training and insufficient resources [29].

Despite some advancements, the distribution of palliative care services in France remains heterogeneous (https://www.igas.gouv.fr/Les-soins-palliatifs-et-la-fin-de-vie-a-domicile.html), as in other European countries [30, 31]. According to WHO, to improve equitable access to palliative care services, emphasis is given to a primary health care approach. In the context of limited specialist palliative care resources available in the community, it is often GPs who provide and co-ordinate palliative and end-of-life care in collaboration with community-based support services [16]. Numerous studies in various countries have identified a lack of knowledge and utilization of palliative care services among GPs [29, 32, 33]. In a survey from the United Kingdom, many GPs reported variability in the availability of specialist palliative care services, particularly a lack of local hospice beds [34].

To address these gaps, our study aimed to describe the knowledge, practices, and perceptions of GPs regarding palliative and end of life care in mainland France.

## Materials and methods

### Study design and population

Between November 2020 and November 2021, we conducted a prospective cross-sectional study among a sample of GPs in the Sentinelles network. The Sentinelles network (www.sentiweb.fr) is a French epidemiologic surveillance system based on primary care in mainland France. This network also provides Sentinelles GPs the opportunity to participate in epidemiological studies beyond their surveillance activities.

The Hestia study group is a group of researchers (general practitioners, internal medicine specialist, palliative doctor, epidemiologist) whose aim is to carry out palliative care studies in France through the Sentinelles network. These studies have been proposed to Sentinelles GPs, and 135 accepted to participate. We first built a national, descriptive study in metropolitan France to describe the palliative care practices of French GPs.

To ensure a representative sample geographical distribution mirroring GPs across mainland France, we stratified the country into five major interregions: Île-de-France (Greater Paris area), Northeast, Northwest, Southeast, and Southwest. The selection of our sample composition of GPs was proportionate to the geographical distribution of French GPs within each of these major interregions.

### Data collection

A self-report questionnaire was developed by the Hestia study group. The questionnaire was tested with ten GPs between February and May 2019 through a qualitative study involving semistructured interviews. Adjustments were made based on the results of this initial study, and questions pertaining to the period "Since the COVID-19 pandemic" were subsequently incorporated.

The data were collected between November 2020 and June 2021. GPs had the option to respond to questions either on paper or electronically through an electronic case report form (eCRF). The eCRF design and distribution were executed using REDCap® Software version 8.4.3. To increase the response rate and address missing data, reminders were dispatched twice via email (December 2020 and April 2021) and three times via phone (December 2020, March 2021, and June 2021).

### Questionnaire components

The questionnaire covered:

- Sociodemographic characteristics of the GPs: age, sex, type of practice, and exercise zone (according to the French National Institute of Statistics and Economic Studies, Insee, https://www.insee.fr);
- Background on palliative care training;
- Perception of available palliative care services in their exercise zone and their utilization before the COVID-19 pandemic;
- Self-assessment of knowledge and competencies related to palliative care announcements, French end-of-life law, pain management, and sedation practices before the COVID-19 pandemic.

To validate GPs' statements about palliative care services against actual availability, we researched existing services. We utilized:

- The directory on the website of French society for support and palliative care (SFAP, www.sfap.org);
- Google Maps (www.google.fr/maps) was used to verify resource availability when the SFAP could not confirm the existence of services;
- Direct phone calls were made to care facilities (hospital or drugstore) in the GP exercise zone to ascertain resource availability where other methods were inconclusive.

### Data processing and analysis

For analytical and clinical purposes, several variables were categorized as follows:

- Type of exercise: categorized as either solo- or group-based (group, home and health center, multisite center);
- Palliative care training: a composite variable derived from responses to independent training-related questions. Categories included no training, initial training only, continuing training only, initial and continuing training, and specialized training (e.g., university diplomas, interuniversity diplomas in palliative care, complementary specialized studies diplomas in pain medicine or palliative medicine, university diplomas, or other specific qualifications in pain management);
- Professional experience in palliative care: indicated by having worked in a department practising palliative care;
- Classification of palliative care services: Grouped into homecare expertise services and care providers (hospitalization at home, home nursing services, and health services providers), hospital expertise or hospitalization services dedicated to palliative care (palliative care unit and identified palliative care beds), and coordination expertise (territorial support platform or coordination support facilities, palliative care mobile team, and palliative care networks).
- Physicians’ assessment of palliative care: GPs were asked to evaluate whether proposals at different stages of an illness fell under palliative care, rated on a scale from 0 (disagreement) to 5 (total agreement);
- Perception ratings of knowledge: GPs' perceptions of their knowledge regarding French end-of-life law (Claeys Leonetti law), trusted third parties, and advance directives were rated on a scale from 0 (unknown) to 5 (very well known);
- Comfort level ratings: GPs' comfort levels regarding pain management, deep and continuous sedation for pain relief, and indications were rated on a scale from 0 (not at all) to 5 (totally).

For these last three questions (assessment, knowledge, comfort), the responses were recoded into categories: 0–1 (low), 2–3 (medium), and 4–5 (high).

The descriptive analysis included all the collected variables. Categorical variables are presented in terms of numbers and proportions, while continuous variables are expressed as medians and standard deviations with minimum and maximum values. Outliers were meticulously examined and corrected if necessary. Details regarding missing data can be found below each respective table and figure.

All analyses were conducted using R Software version 3.6.3.

## Results

Out of the 135 voluntary participating GPs, 91% (*n* = 123) responded to the questionnaire.

### Participants demographics and location

The median age of the GPs was 48 years, and less than half (47%) were women (Table 1). Slightly more than a quarter (29%) of the GPs practiced in rural areas, and approximately one third (30%) worked alone.

**Table 1:** Demographics characteristics and location of general practitioners

	General practitioners n (%), *N*= 123
**Age:** median±SD [min; max] years old	48±12.6 [29; 74]
**Age categories (years old)**	
<50	66 (54)
≥50	57 (46)
**Sex**	
Woman	58 (47)
Man	65 (53)
**GPs exercise** ** type**	
Alone	37 (30)
Group	86 (70)
**GPs exercice** **zone**	
Rural	35 (28)
Urban	88 (72)
**GPs localisation in major** ** interregions**	
Île-de-France (Greater Paris area)	17 (14)
Northeast	27 (22)
Northeast	28 (23)
Southeast	27 (22)
Southeast	24 (19)

*SD* standard deviation, *GPs* general practitioners; there were no missing data for any of the variables

### Participants training and experience in palliative care

More than half of the GPs in the sample (59%) have received minimal training in palliative care, and a majority (84%) lack professional experience in palliative care (Table 2).

**Table 2:** General practitioners’ training and experience in palliative care

	General practitioners n (%), *N*= 123
**GPs training level in palliative care**	
None	19 (16)
Initial only	47 (38)
Continuing only	26 (21)
Initial and continuing	20 (16)
Specialized	20 (16)
**Professional experience acquired by GPs in a palliative care service**	
No	104 (84)
Yes	19 (16)

*GPs* general practitioners; there were no missing data for any of the variables

### General practitioners’ knowledge of the French end-of-life law (Claeys Leonetti law)

**Fig. 1:**
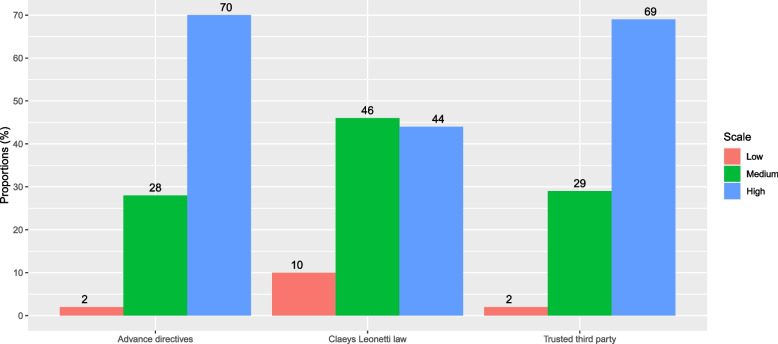
General practitioners’ knowledge of the French end of life law (Claeys Leonetti law). Responses to the question “How would you rate your knowledge of the following devices before the COVID-19 pandemic? : 1-end-of-life law (Claeys Leonetti law), 2-trusted third party, and 3-advance directives” were graded on Likert scales ranging from 0 to 5 (0: absent; 5: very good) and were recoded into categories: 0-1 (low), 2-3 (medium), and 4-5 (high). The histograms show the percentage of general practitioners responding to each provision of the law (total *n* = 123). The figures above the bars indicate the percentage for each response category: low (red), medium (green), and high (blue). There were no missing data for any of the variables

A remarkable percentage of GPs reported a high level of knowledge of the provisions of the law on end of life, particularly in relation to advance directives (70%) and trusted third parties (69%). However, a tenth of them reported low knowledge of the law as a whole (Fig. 1).

### General practitioners’ perception about identifying patient who need palliative care

**Fig. 2:**
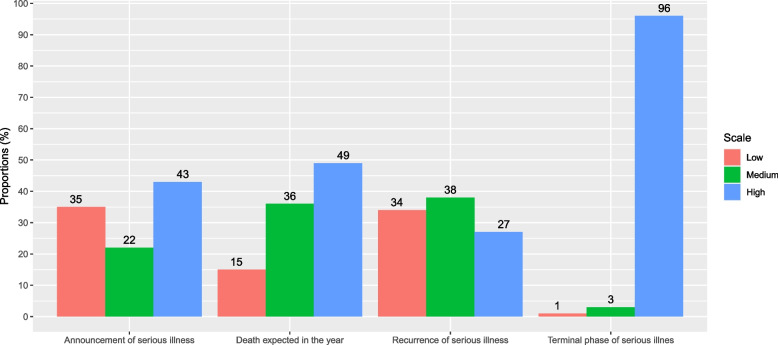
General practitioners’ perception about identifying patient who need palliative care. Responses to the question “In your opinion, at what point(s) can patient care be qualified as palliative?: 1-when a serious, life-threatening illness is announced, 2-in the event of a recurrence of a serious illness, 3-in the terminal phase of a serious illness, and 4-as soon as I will not be surprised that my patient could die within the year” were graded on Likert scales ranging from 0 to 5 (0: disagree; 5: completely agree) and were recoded into categories: 0-1 (low), 2-3 (medium), and 4-5 (high). The histograms show the percentage of general practitioners responding to each situation (total *n* = 123). The figures above the bars indicate the percentage for each response category: low (red), medium (green), and high (blue). There were no missing data for any of the variables

The terminal phase of a serious illness was recognized by most GPs as the point at which care could be qualified as "palliative" (96%). For other scenarios, such as a foreseeable death within the year and the announcement of a serious and life-threatening disease, fewer than half of the GPs perceived the care as "palliative" (49% and 43%, respectively). In the case of a recurrent serious illness, 27% of the GPs qualified the care as palliative (Fig. 2).

### General practitioners’ confidence about palliative and end of life care practices

**Fig. 3:**
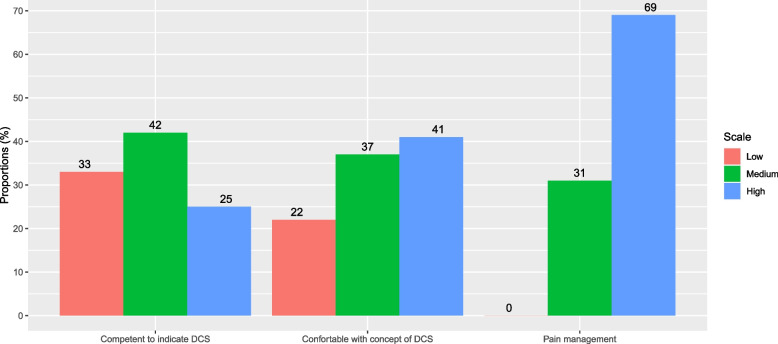
General practitioners’ confidence about palliative and end of life care practices. DCS: deep and continuous sedation. Responses to the question “Before the COVID-19 pandemic, did you feel?: 1-comfortable with pain management, 2-comfortable with the concept of deep and continuous sedation for pain relief, and 3-competent to indicate deep and continuous sedation for pain relief” were graded on Likert scales ranging from 0 to 5 (0: not at all; 5: totally) and were recoded into categories: 0-1 (low), 2-3 (medium), and 4-5 (high). The histograms show the percentage of general practitioners responding to each situation (total *n* = 123). The figures above the bars indicate the percentage for each response category: low (red), medium (green), and high (blue). There were no missing data for any of the variables

The majority of the participants were comfortable with pain management (69%), 41% were comfortable with the concept of deep and continuous sedation. However, one third was not competent to establish the indication of deep and continuous sedation for pain relief (Fig. 3).

### Availability, knowledge, and utilization of palliative care services according to general practitioners

Almost all the participating GPs (99%) reported the availability of home palliative care expertise, 89% reported coordination palliative care expertise, and 83% reported hospital palliative care expertise. Of the 117 GPs (95%) who reported having hospital or coordination expertise available, 81% had both. Regarding specific services, 95% of all GPs reported having hospitalization at home available, 70% had access to palliative care units within a 40 km radius, and 67% had a palliative care mobile team. Prior to the COVID-19 pandemic, 94%, 46%, and 53% of all GPs had utilized these palliative care services, respectively (Table 3). Thirty percent (*n* = 37) reported the absence of a palliative care unit within a 40 km radius, and 52% (*n* = 64) did not have identified palliative care beds. Among them, more than a third (14/37) and a third (20/64) were unaware of the existence of palliative care units and identified palliative care beds, respectively (Table 3). These two services were the least familiar to the GPs.

**Table 3:** Palliative care resource mobilization by general practitioners: in relation to service availability, their knowledge, and their utilization^a^, *N* = 123

	**Services reported available by GPs** **n (%)**	**Check availability**^**b**^ **n (%)**	**Use of services**^**c**^ **n (%)**
**General ambulatory services**			
Hospitalization at home	117 (95)	120 (98)	116 (94)
Home nursing service	112 (91)	115 (94)	105 (85)
Service providers	110 (89)	111 (90)	95 (77)
Mobile geriatric and gerontopsychiatric team	74 (60)	75 (61)	61 (50)
No outpatient services	1 (1)	1 (1)	-^d^
**Specialized palliative care services**			
Palliative care units within 40 km radius	86 (70)	100 (81)	56 (46)
Palliative care mobile team	82 (67)	88 (72)	65 (53)
Identified palliative care beds	59 (48)	79 (64)	34 (28)
Palliative care networks	55 (45)	61 (50)	-^d^
Territorial support platform or coordination support system	40 (33)	42 (34)	24 (20)
No specialized services	6 (5)	1 (1)	0 (0)

*GPs* General practitioners; there were no missing data for any of the variables

^a^Only responses equal to “yes” are presented in this table

^b^Checking the availability of all services reported by the GPs by the research team

^c^Prior to the COVID-19 pandemic

^d^Data not collected

## Discussion

### Main findings

In light of several studies demonstrating that GPs lack both training in palliative and end-of-life care, as well as knowledge and utilization of palliative care services, our study provides additional data on the practices of French GPs in this context. Our study reveals that more than half of French GPs have minimal training in palliative care and a majority lack experience in palliative care service. Overall, they are familiar with French end-of-life legislation, particularly the mechanisms of advance directives and the designation of a trusted third parties. It also reveals that more than two-thirds of the GPs expressed comfort with pain management, while only a quarter felt competent to indicate deep and continuous sedation for pain relief. Regarding the awareness and use of palliative care services near their practice area, outpatient services were the most recognized and utilized by GPs compared to hospital-based services. Although these results are encouraging, they highlight the need for continued training of GPs in palliative care and the promotion of coordination between hospital and outpatient teams. These findings are particularly significant in the French context, where midazolam is now available to GPs for sedation since 2021.

### General practitioners training in palliative and end of life care

Regarding GPs training in palliative care, our study corroborates previous research findings suggesting the need of better training for GPs. In our study, only approximately 2 out of 5 GPs reported receiving initial training in palliative care. Similar results were found in other studies in Germany, in Australia, and in the United States [23, 35, 36]. However sixteen percent of GPs in our study reported having never received training in palliative care, similar to the 13% reported in a study from the United Kingdom [37] but less than 50% reported in a Dutch study [6]. In terms of knowledge level, studies indicate that a majority of GPs express a desire to improve their knowledge of palliative care (for example, 80% in Norway and 60% in Romania) [18, 38]. Thus, providing palliative care training that aligns with GPs’ clinical practice and learning preferences could improves their knowledge, skills and confidence [26, 27, 39]. For example, some Australian GPs perceived palliative care as a natural extension of primary care and indicated that best practice palliative care mainly requires experiential knowledge and good communication skills, rather than specialised medical knowledge [15]. In addition, identifying GP trainees perceived educational needs could help to inform the development of a robust postgraduate palliative care curriculum suitable for trainees in practice [24].

### General practitioners’ knowledge, confidence and perceptions about palliative and end of life care

There are notable differences in GPs' knowledge, confidence, and perceptions of palliative and end-of-life care across different regions worldwide, as compared to other studies.

Our study reveals that just over half of French GPs had moderate or low knowledge of end of life law. This contrast with the results from an Australian study among professionals working in the elderly care sector, where 4 out 5 professionals reported having moderate or low knowledge of end-of-life law provisions [40].

Regarding pain management and sedation at the end of life, the literature, including our study, shows that implementing deep sedation is more challenging than managing pain. For example, in the United States, Switzerland and Denmark, studies shows that 78%-88% of GPs felt comfortable with pain management or discussions related to advance directives [17, 36, 41]. However, in Norway, a quarter of GPs did not feel comfortable administering palliative care treatments [18]. These findings align with our study and indicate a need for additional training in deep and continuous sedation for GPs. Beyond these implications for practice, these findings have a significant impact on patient care. The reluctance of GPs to initiate deep and continuous sedation is concerning, particularly in light of potential changes in end-of-life legislation in France and the increasing need for home based palliative care due to the aging population worldwide. This reluctance may result in many patients not receiving necessary sedation. However, a recent study shows that midazolam is the most commonly used drug for sedation (85.9%) in French palliative care service [42]. Improving access to midazolam in primary care and providing GPs with adequate training in sedation techniques could help address this issue. The practice and frequency of deep and continuous sedation vary across countries [43, 44]. In nationwide studies the frequencies of deep and continuous sedation in deceased persons varied from 3% in Denmark in 2001 to 18% in the Netherlands in 2015 [45]. This align with the results from a recent French study among palliative care services that show a prevalence of any type of sedation of approximately 3% [42]. These differences in the practice of the sedation can be explained by the varying recommendations and legalization of euthanasia in each country [44, 46].

Regarding the identification of patients in need of palliative care, Dutch GPs reported that a combination of several signals, often subtle and not explicit, made them identify a need for palliative care: signals from patients (increasing care dependency and not recuperating after intercurrent diseases) and signals from relatives or reports from medical specialists [22]. However, our study focused solely on somatic signals, limiting the ability to make direct comparisons with these results. Nevertheless, it provides insight into how GPs identify patients in need of palliative care, which is crucial for the timely initiation of palliative care services. This identification is even more important since many GPs initiate palliative care early, at a stage when hospitalization in specialized facilities is not yet necessary. Therefore, it is essential for these GPs to be aware of and utilize outpatient palliative care services.

### Availability, knowledge, and utilization of palliative care services according to general practitioners

The literature including our study highlights the heterogeneous knowledge and use of palliative care services among GPs in different countries.

In France, the structuring of palliative care at both clinical and educational levels has recently occurred. Our study revealed a lower awareness among GPs regarding the availability of certain palliative care resources, particularly hospital-based services (palliative care units and identified palliative care beds). Half of our sample of GPs had utilized these services during the study period. In a French study, less than third percent of deceased individuals in 2013 (61% of all dead in France) received hospital-based palliative care either at the time of death or during the year preceding death [47]. However, this study did not account for palliative care managed by GPs, suggesting a potentially low level of utilization by GPs, given that many patients died in hospitals. In our study, hospitalization at home was the most well-known and utilized resource by GPs. This difference in use can be explained in part by the fact that GPs consider hospitalization at home indispensable and use it as a solution to social isolation during palliative care, according to a French qualitative study [48]. Worldwide, GPs' knowledge and use of palliative care services vary across countries. In England, for example, more than a third of GPs were unaware of the availability of palliative care services during evenings, nights, or weekends [32]. In Australia, a study shows that a third of GPs were unaware of hospital palliative care services, or home palliative care services [29]. Like our results, in a Dutch study, most GPs reported that they sometimes or often involved palliative home care teams [6]. A European study conducted in Belgium, Spain, Italy, and the Netherlands showed that GPs used palliative care services for less than half of their patients in the last three months of life [33]. In France, although palliative care resources are available, their awareness is incomplete, and their mobilization by GPs depends on resource availability, their knowledge and their utilization by healthcare professionals.

In addition to training, the coordination and communication of the various systems remain essential.

### Strengths and limitations

To the best of our knowledge, this study is among the first in France to describe GP practices related to palliative care. Rigorous follow-ups with GPs were conducted to ensure comprehensive data collection without any omissions. However, a notable limitation is the potential for selection bias, which may constrain the generalizability of our findings. The participating GPs constitute a relatively small population drawn from a research network, introducing a potential source of bias. In an effort to mitigate this bias, we sought to standardize our sample distribution by aligning it with that of GPs in mainland France, applying proportional representation across each interregion. Despite our standardization efforts, our sample of GPs was over-represented in the Northwest (23% *versus* 19%) and younger (48 years *versus* 52 years) compared to GPs in mainland France [49]. It is important to acknowledge that self-assessments by GPs of their knowledge and practices could influence the interpretative scope of our results.

## Conclusions

French GPs exhibit awareness of the palliative care services accessible within their practice zones. Given that French GPs are now authorized to prescribe midazolam in primary care and considering potential future changes in French end-of-life law, it is crucial that they receive additional training in deep and continuous sedation which has been the gap identified by GPs. An observational study with a substantial sample size would be valuable for comprehensively assessing French GPs' practices in the realm of palliative care.

### Supplementary Information

Supplementary Material 1.

## Data Availability

The datasets generated and/or analysed during this study are not publicly available due to the policy of the French Data Protection Authority, but are available from the corresponding author upon reasonable request.
