# Supplementary material for: General practitioners and palliative care practices: a better knowledge of specific services is still needed
Source: BMC Health Serv Res. 2024 Jul 23;24:832. doi: 10.1186/s12913-024-11266-8 (PMC11264423; doi:10.1186/s12913-024-11266-8)
Supplement: Supplementary file 1 — Supplementary Material 1. [file 12913_2024_11266_MOESM1_ESM.docx]

Inclusion number :

Physician questionnaire – Hestia study

***I – Your sociodemographic characteristics***

| 1. | How old are you? | ..….. years |
| --- | --- | --- |
| 2. | What is your gender? | 🞏 Female  🞏 Male |
| 3. | How old were you when you first set up? | ……. years |
| 4. | What kind of practice do you have? | 🞏 Alone  🞏 In group  🞏 In a care home (private structure)  🞏 In health center (public structure)  🞏 multi-site center |
| 5. | What is your exercise zone? | 🞏 Rural  🞏 Semi-rural  🞏 Urban |

***II - Training received***

| 6. | What training have you received in palliative care? (one or more answers)   - No training - Initial training - Continuing training - Have worked in a department practicing palliative care - University diploma or Inter-university diploma in palliative care - Complementary specialized studies diploma in pain medicine or palliative medicine - University diploma or other specific qualification in pain management - Others (specify) ………………………………………. |
| --- | --- |

***III – Palliative services and resources available to you***

1. Which of these resources **do you have** close to your place of work? (one or more answers)

- Hospitalization at home
- Home nursing service
- Mobile geriatric and gerontopsychiatric team
- Services providers
- None of these resources

1. Which of these palliative resources **do you have** close to your place of work? (one or more answers)
   - Territorial support platform or coordination support system
   - Palliative care mobile team
   - Identified palliative care beds
   - Palliative care units within 40 km radius
   - Palliative care networks
   - None of these resources
2. Which of the following resources have you worked with before the Covid-19 pandemic? (one or more answers)

| - - - Hospitalization at home |  |
| --- | --- |
| - - - Home nursing service |  |
| - - - Mobile geriatric and gerontopsychiatric team |  |
| - - - Services providers |  |
| - - - Territorial support platform or coordination support system |  |
| - - - Palliative care mobile team |  |
| - - - Identified palliative care beds |  |
| - - - Palliative care units within 40km radius |  |
| - - - None of these resources |  |

1. In your opinion, at what point(s) can patient care be qualified as palliative? (circle on a scale from 0 to 5, 0: disagree; 5: completely agree)

| - - - When a serious, life-threatening illness is announced | 0 | 1 | 2 | 3 | 4 | 5 |
| --- | --- | --- | --- | --- | --- | --- |
| - - - In the event of a recurrence of a serious illness | 0 | 1 | 2 | 3 | 4 | 5 |
| - - - In the terminal phase of a serious illness | 0 | 1 | 2 | 3 | 4 | 5 |
| - - - As soon as I will not be surprised that my patient could die within the year | 0 | 1 | 2 | 3 | 4 | 5 |

1. How would you rate your knowledge of the following devices before the Covid-19 pandemic? (circle on a scale from 0 to 5, 0: absent; 5: very good)

| - - - End-of-life low (Claeys  Leonetti law) | 0 | 1 | 2 | 3 | 4 | 5 |
| --- | --- | --- | --- | --- | --- | --- |
| - - - Trusted third party | 0 | 1 | 2 | 3 | 4 | 5 |
| - - - Advance directives | 0 | 1 | 2 | 3 | 4 | 5 |

1. Before the Covid-19 pandemic, did you feel? (circle on a scale from 0 to 5, 0: not at all; 5: totally)

| - - - Comfortable with pain management | 0 | 1 | 2 | 3 | 4 | 5 |
| --- | --- | --- | --- | --- | --- | --- |
| - - - Comfortable with the concept of deep and continuous sedation for pain relief | 0 | 1 | 2 | 3 | 4 | 5 |
| - - - Competent to indicate deep and continuous sedation for pain relief | 0 | 1 | 2 | 3 | 4 | 5 |
